# Spindle-like MRI-active europium-doped iron oxide nanoparticles with shape-induced cytotoxicity from simple and facile ferrihydrite crystallization procedure†

**DOI:** 10.1039/c9ra10683a

**Published:** 2020-02-18

**Authors:** Afanasy V. Lunin, Ilya L. Sokolov, Ivan V. Zelepukin, Ilya V. Zubarev, Maria N. Yakovtseva, Elizaveta N. Mochalova, Julian M. Rozenberg, Maxim P. Nikitin, Eugene L. Kolychev

**Affiliations:** Moscow Institute of Physics and Technology (National Research University) 9 Institutskiy per., Dolgoprudny Moscow 141700 Russia; Shemyakin–Ovchinnikov Institute of Bioorganic Chemistry, Russian Academy of Sciences Ulitsa Miklukho-Maklaya, 16/10 Moscow 117997 Russia; Prokhorov General Physics Institute of the Russian Academy of Sciences 38 Ulitsa Vavilova St. Moscow 119991 Russia eugene.kolychev@gmail.com

## Abstract

Nanoparticles (NPs) that can provide additional functionality to the nanoagents derived from them, *e.g.*, cytotoxicity or imaging abilities, are in high demand in modern nanotechnology. Here, we report new spindle-like iron oxide nanoparticles doped with Eu^3+^ that feature magnetic resonance imaging (MRI) contrasting properties together with shape-related cytotoxicity (unusual for such low 2.4% Eu content). The NPs were prepared by a novel procedure for doping of iron oxide nanoparticles based on the crystallization of amorphous ferrihydrite in the presence of hydrated europium(iii) oxide and were thoroughly characterized. Cytotoxicity of low Eu-doped spindle-like hematite nanoparticles was confirmed by MTT assay and further studied in detail by imaging flow cytometry, optical and electron microscopies. Additionally, enhancement of MRI contrast properties of NPs upon doping with europium was demonstrated. According to the MRI using mice as an animal model and direct inductively coupled plasma mass spectrometry (ICP-MS) ^153^Eu biodistribution measurements, these particles accumulate in the liver and spleen. Therefore, NPs present a novel example of a multimodal component combining magnetic imaging and therapeutic (cytotoxic) abilities for development of theranostic nanoagents.

## Abstract 2

Novel MRI active spindle-like nanoparticles prepared by a facile procedure display cytotoxicity due to synergistic combination of shape and europium content.

## Graphical abstract

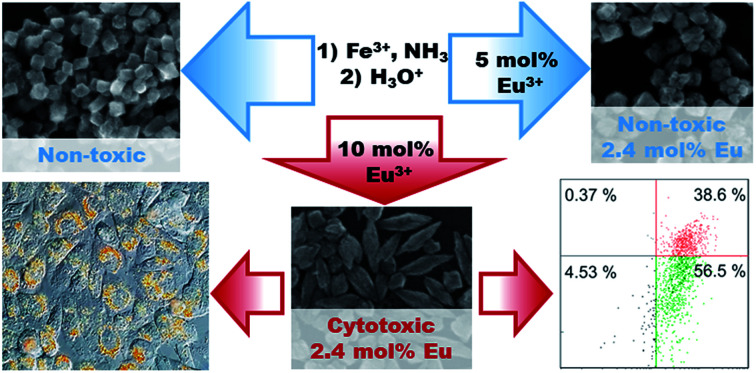

## Introduction

One of the rapidly developing applications of nanostructured materials is the construction of nanoagents capable of detection and selective elimination of pathogens including bacterial infections and cancer cells.^1–4,56^ Such agents are usually constructed using a nanoparticle as a base. This particle may have no function but bearing surface-modified layers of reagents for selective detection, imaging, and killing pathogen cells.^5^ Therefore, the nanoparticle plays the role of an inert carrier. Meanwhile, the nanoparticles (NPs) can bring some functionality to the nanoagents, *e.g.*, capability of optical or magnetic detection, or be cytotoxic on their own.^6^ In the latter case, the surface modification can be aimed only at the targeted delivery of the particle and does not require a more complex modification for binding of cytotoxic agents. Moreover, the design of nanoagents may be more convenient if the nanosized core combines the imaging and therapeutic functions. An attractive way for facile preparation of the multifunctional nanoparticles of this type is doping them with an agent bringing an additional modality. Such modifications mostly require a small amount of doping reagent, which provides alteration of the original properties of undoped nanoparticles.^7–10^

For example, doping of NPs with europium can provide a number of functionalities: (i) allow precise highly-sensitive detection of the particles *ex vivo* with inductively coupled plasma mass spectrometry (ICP-MS) due to absence of this metal in mammal tissues; (ii) make NPs fluorescent, which is highly useful for visualization NPs *ex vivo* and *in vivo*; (iii) enhance MRI-contrasting properties of the NPs.

Iron oxide nanoparticles are widely used to design biocompatible nanoagents, which additionally could be used as contrast agents for MRI imaging.^11–13^ The NPs of hydrous ferric oxides such as goethite and hematite have a large specific surface area; the surface is hydrophilic and can be modified by adsorbing various cations and anions, which can introduce a novel functionality to the particles.^14–17^ However, the adsorption of the metal ions by these particles is usually a reversible process. Therefore, the metal ions can easily leak into the solution from the surface of the nanoparticles.^18–21^

Recently, we reported on the synthesis of well-defined crystalline hydrous ferric oxide particles obtained by acid-mediated crystallization of ferrihydrite.^22^ Crystallization of ferrihydrite together with another amorphous metal oxide may produce the nanoparticles, in which the dopant is fairly distributed not only on the surface but also throughout the whole volume of the nanocrystal. Such nanoparticles would not lose the doping ion even if their surface is dissolved or destroyed.

Here, we report on the novel synthesis of spindle-like iron oxide nanoparticles doped with Eu^3+^ by crystallization of ferrihydrite in the presence of hydrated europium oxide, and preliminary investigation of their cytotoxic and MRI contrasting properties. Moreover, the presented data have shown that the toxicity is the result of not only heavy metal content but also a combination of shape and europium doping.

## Experimental

### Starting materials

Sigma-Aldrich (USA): FeCl_3_·6H_2_O (>99%), CaCl_2_·6H_2_O (>98%), carboxymethyl-dextran sodium salt (CMD, >90%, cat. 86524-100G-F), fluorescein isothiocyanate isomer I (FITC, >90%), paraformaldehyde (95.0–100.5%), glutaraldehyde (grade I), sodium cacodylate trihydrate (BioXtra, >98%), osmium tetroxide (>99%), PBS (>99%) and HEPES (>99.5%) buffers; Alfa Aesar (Germany): EuCl_3_·6H_2_O (>99.99%); Biotium (USA): propidium iodide (PI) (>95%); BioLegend (USA): Purified Annexin V (cat. 640901); Dia-M (Russian Federation): nitric acid (70%, w/w), methylthiazolyldiphenyl-tetrazolium bromide (MTT, >98.5%), aqueous ammonia (25%, w/w); AppliChem (Germany): Triton X-100 (Molecular biology grade); Rushim (Russian Federation): dimethyl sulfoxide (DMSO, 98%). All commercially available reagents were used as received. Saline solution was of medicine grade. Milli-Q water (Merck Millipore, USA) was used in the preparation of aqueous solutions.

### NPs synthesis

The different volumes of 0.3 M aqueous solution of EuCl_3_ (0 μL, 50 μL, 100 μL, and 200 μL) were added to 1 mL of 0.3 M aqueous solution of FeCl_3_. The resulting mixtures were treated with 125 μL of 25% aqueous ammonia and homogenized in Vortex, followed by incubation at 90 °C for 2 h. After the reaction was complete, the precipitate was washed by centrifugation and mixed with 1.0 mL 0.6 M nitric acid. The resulting mixture was stirred overnight at room temperature and carefully washed with Milli-Q water. CMD coating was performed as follows: 150 μL of NPs with a concentration of 35 g L^−1^ were mixed with 150 μL of 10% (w/v) aqueous solution of CMD, incubated overnight at 90 °C and washed 2 times by centrifugation with Milli-Q water.

### NPs characterization

The particle hydrodynamic diameters were determined using a Photocor Complex (Photocor Ltd., Russia). All the measurements were performed in Milli-Q water at room temperature. Scanning electron microscopy images of NPs were obtained with a Tescan MAIA3 microscope (Tescan, Czech Republic) at an accelerating voltage of 10 kV. The samples were diluted down to appropriate concentrations, placed onto a silicon wafer and then air-dried. *ζ*-potential of the nanoparticles was measured by electrophoretic light scattering using a Zetasizer Nano ZS (Malvern Instruments, UK). The measurements were performed in 10 mM NaCl solution at room temperature. FTIR spectra were collected with a Nicolet iS50 Spectrometer (Thermo Fisher Scientific, USA). The nanoparticle suspensions were air-dried at 60 °C. Raman spectra were measured with a Confotec MR 350 Raman laser scanning confocal microscope (Sol instruments, Belarus) at a 100× magnification. The nanoparticle suspension was dropped onto a borosilicate glass coverslip and air-dried. A 633 nm laser (40 mW) was used for excitation. X-ray diffraction (XRD) patterns were collected with a Rigaku diffractometer with CuKα radiation (SmartLab, Japan). The nanoparticle suspensions were air-dried at 60 °C. Transmission electron microscopy energy-dispersive X-ray (TEM-EDX) analysis was performed on a 200 kV JEOL JEM 2100 TEM (Tokyo, Japan). Relaxivities were calculated using relaxation times (water solution, RT), measured on a 0.23 T Minispec Analyzer MQ10 (Bruker, USA). Magnetization measurements were performed on a vibrating sample magnetometer VSM 7410 (Lake Shore Cryotronics, USA).

### Animals

Male C57BL/6 mice of 27–32 g weight were used for experiments. All procedures were carried out in compliance with the National Institutes of Health (NIH) Guide for the Care and Use of Laboratory Animals and were approved by the Institutional Animal Care and Use Committee of Shemyakin-Ovchinnikov Institute of Bioorganic Chemistry.

### Magnetic resonance imaging

A mouse was injected with 1 mg of NPs in 100 μL of 150 mM NaCl. After 3 h, the animal was sacrificed by CO_2_ inhalation, and magnetic resonance imaging (MRI) was immediately performed using an ICON 1T MRI system (Bruker, USA) with the mouse whole-body volume RF coil. The following common parameters were used for the sequences: for the RARE sequences: RARE factor = 1, TR = 3000 ms, TE = 40 ms, resolution of 156 μm, FOV = 80 × 30 mm, 4 slices per scan, slice thickness of 1 mm; for the FLASH sequences: flip angle = 30°, TR = 300 ms, TE = 10 ms, resolution of 156 μm, FOV = 80 × 30 mm, 4 slices per scan, slice thickness of 1 mm.

### ICP-MS analysis

Inductively coupled plasma mass spectrometry (ICP-MS) measurements were performed using a NexION 2000 (PerkinElmer, USA) mass spectrometer. Mouse organs were collected immediately after the MRI measurements. Each organ was weighed and digested with 4 parts of concentrated nitric acid for 15 min at 65 °C. The nanoparticles were also dissolved in concentrated nitric acid. The mixtures were diluted 10 times with water and used for the measurements. ^153^Eu peak was used for analysis. To calculate the share of NPs in an organ, we used Formula S1.†

### MTT assay

For the cell viability MTT test, Chinese hamster ovary (CHO) cells were seeded in 10% fetal bovine serum in Dulbecco's Modified Medium/F12 24 h before the experiment. 1000 cells per well were incubated for 24 h, 48 h, and 72 h in 200 μL of the medium with NPs in the following concentrations: 500, 160, 50, 16, 5, and 0 μg mL^−1^. After that, the medium was replaced by 100 μL of 0.5 mg mL^−1^ MTT solution in the media and incubated for 30 min to form formazan crystals. Then, the medium was removed, and the samples were homogenized in 100 μL of dimethyl sulfoxide. The samples were transferred to a different plate, and the solution absorption was measured by a spectrophotometer at 570 nm.

### SEM of CHO cells

Cell cultures for scanning electron microscopy (SEM) were cultured on a cover glass prior to the formation of a monolayer. The cells were fixed in 2% paraformaldehyde and 2.5% glutaraldehyde in cacodylate buffer (0.1 M, pH 7.2, according to Karnovsky) with 5% sucrose, and then postfixed in 2% osmium tetroxide water solution. The material was dehydrated in alcohol and acetone, and dried at a critical point in a K850 Critical Point Dryer (Quorum Technologies, UK). A 15 nm gold/palladium layer was deposited onto the surface of the samples in a Q150T Plus metal sputtering device (Quorum Technologies, UK). The cells were studied in an AURIGA FIB-SEM workstation scanning electron microscope (Carl Zeiss & MT, Germany) with a SE detector in the 50–50 000 magnification range.

### Imaging flow cytometry

Purified Annexin V was labeled with FITC according to the manufacturer's protocol in 1 : 60 molar concentration ratio of Annexin V : FITC. The CHO cells (30 000 per well) were seeded onto 6-well plates and cultured overnight, followed by incubation with 50 μg mL^−1^ NPs in 10% fetal bovine serum in Dulbecco's Modified Medium/F12. Then, the cells from each well were harvested and resuspended in 50 μL of annexin V binding buffer (10 mM HEPES pH 7.4, 140 mM NaCl, and 2.5 mM CaCl_2_ solution). After 10 min incubation with 0.5 μg mL^−1^ Annexin V-FITC in PBS and followed 5 min incubation with 0.25 μg mL^−1^ PI in PBS, the cells were immediately processed with an imaging flow cytometer ImageStream X Mark II (Luminex Corporation, USA) using a 40× objective, 488 nm laser (50 mW) for excitation of fluorescence and 785 nm laser (0.2 mW) for side scatter measurements. We gated focused single cells, 1500 events were collected for each sample. For the obtained data, the compensation was applied.

### Optical microscopy

The CHO cells were seeded in imaging dishes (30 000 cells per dish) with coverslip bottom and were incubated overnight in 1.9 mL of 10% fetal bovine serum in Dulbecco's Modified Medium/F12 at 37 °C. Then 100 μL of NPs suspension were added to reach the final NPs concentration of 50 μg mL^−1^. After that, the dishes were incubated for 48 h. Finally, 0.75 μL of Hoechst (10 g L^−1^ in PBS) and 5 μL PI (50 mg L^−1^ in PBS) were added, and the cells were stained for 10 min.

All microscopy images were acquired with a FLUOVIEW FV3000 confocal laser scanning microscope (Olympus Corporation, Japan) using an ×40 objective. For fluorescent imaging, 405 nm (0.75 mW) and 561 nm (0.4 mW) lasers, and cooled GaAsP photomultipliers as detectors were used. To produce differential interference contrast (DIC) images, a MicroPublisher 5.0 RTV camera (Teledyne QImaging, Canada) was used.

### Absorbance measurements

The absorbance measurements were recorded with a ClarioStar microplate reader (BMG LABTECH, Germany) using polystyrene 96-well plates. For UV/Vis spectrum recording, UV-transparent 384-well plates were used.

### Nanoparticle sterilization

For all *in vitro* experiments, NPs were sterilized *via* autoclaving for 20 min at 121 °C in Dulbecco's Modified Medium/F12 (without serum).

### Biochemical serum assay

All the assays were performed using commercial kits. ALT 360 and BIL 100 S were purchased from BIO-LACHEMA-TEST, Czech Republic; LDH-2-OLVEX, AST-RF-OLVEX, and CREATININE-D-OLVEX were purchased from Olvex Diagnosticum, Russian Federation. All the assays were used according to the instructions. To examine mice samples, quantities of the reagents were scaled proportionally.

## Results and discussion

**Fig. 1:**
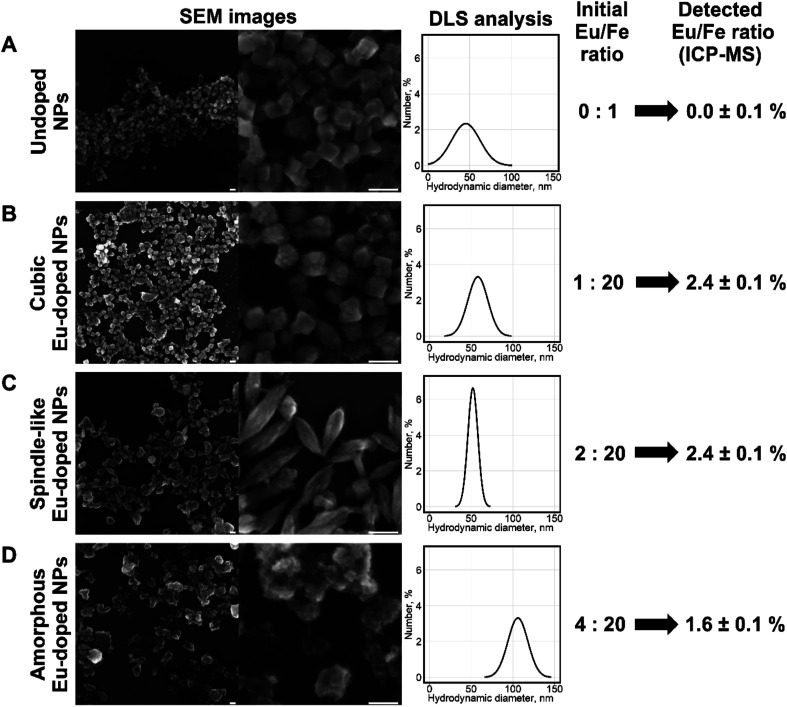
Analysis of nanoparticles prepared from mixtures of iron(iii) chloride with different amounts of europium(iii) chloride: row A: no europium, row B: Eu/Fe molar ratio = 1 : 20, row C: Eu/Fe molar ratio = 1 : 10, row D: Eu/Fe molar ratio = 1 : 5. Two left images in each row show SEM images, scale bar is 100 nm; the plot and scheme in each row show results of DLS and elemental analyses, respectively.

The NPs were prepared by the modified procedure described recently.^22^ Briefly, mixtures of iron(iii) and europium(iii) chlorides were precipitated using aqueous ammonia. The precipitate was resuspended in HNO_3_ and stirred overnight. The resulting NPs containing different amounts of europium were analyzed by scanning electron microscopy (SEM), inductively coupled plasma mass spectrometry (ICP-MS), and dynamic light scattering (DLS). According to SEM (Fig. 1) the morphology of the nanoparticles changed from cubic to less-defined cubic at the Eu/Fe molar ratio of 1 : 20 in the reaction mixture to well-defined spindle-like particles at the Eu/Fe molar ratio of 1 : 10, which is in agreement with the data previously obtained for the hydrothermal synthesis of Gd-doped iron oxide particles.^23^

Further increase in europium concentration (Eu/Fe molar ratio 1 : 5) led to the formation of poorly defined amorphous particles. The DLS analysis showed a slight increase in hydrodynamic diameter from 44 ± 18 to 58 ± 12 and 52 ± 6 nm at the Eu/Fe molar ratios of 1 : 20 and 1 : 10, respectively. Whereas the amorphous particles size drastically increased to 106 ± 12 nm. The ICP-MS analysis showed that europium content in NPs was lower than the amount of europium in the reaction mixture. The highest Eu/Fe molar ratio, which allowed obtaining well-defined uniform nanoparticles, was 1 : 10. Those NPs contained 2.4% europium and were studied in detail. They were used for further investigation of toxicity and MRI-contrasting properties. All the syntheses were carried out at least 10 times for each Eu/Fe ratio to ensure reproducibility.

We believe that europium acts not only as a dopant but also as an inhibitor of the reaction. An increase of Eu/Fe molar ratio causes a decrease in the reaction yield; moreover, if the ratio is 1 : 2, the reaction does not occur. As a result, we suppose that an increased Eu/Fe ratio enhances the europium doping ratio but may somehow interrupt the reaction. These facts explain the maximal doping ratio of 2.4% that is reached at different initial conditions. The effect of europium on the reaction is unclear and needs a detailed investigation.

**Fig. 2:**
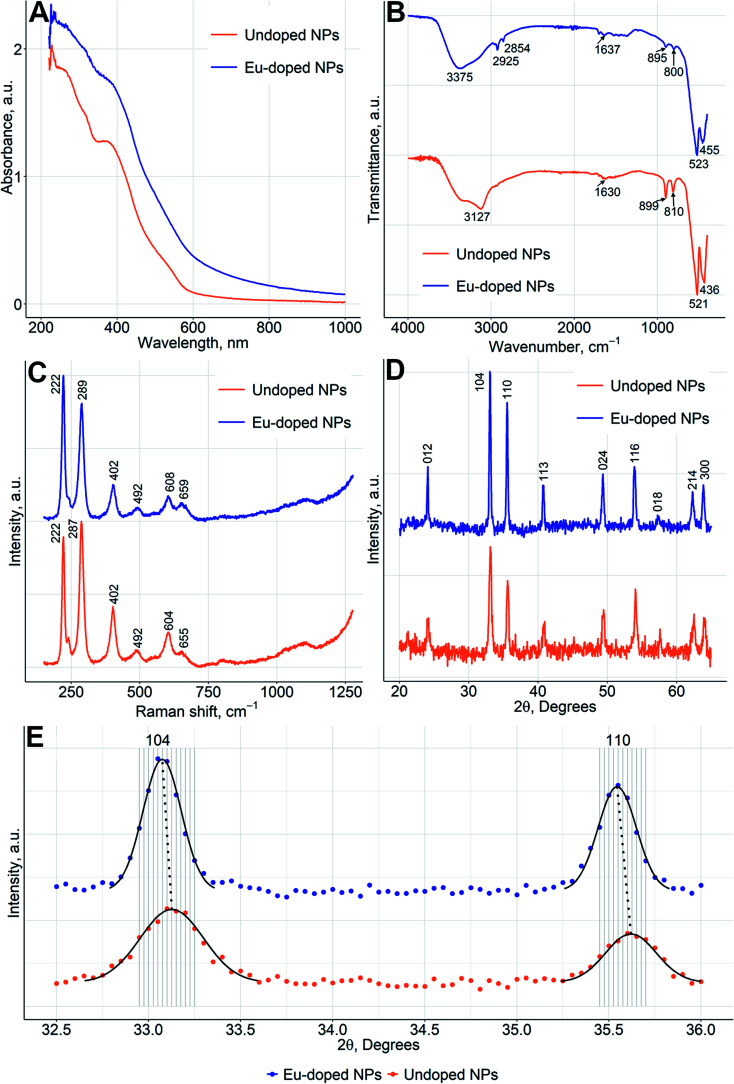
Characterization of the physical properties of the undoped NPs and the spindle-like europium-doped NPs: (A) UV/Vis absorption spectra; (B) FTIR transmittance spectra. Characteristic absorption bands are indicated by arrows; (C) Raman spectra with marked positions of peaks; (D) XRD patterns. Peaks are marked with corresponding Miller indices; (E) XRD patterns at the angular range 32.5–36.0°. The black solid lines present Gaussian peak approximation, the black dotted lines link corresponding peaks maxima.

**Fig. 3:**
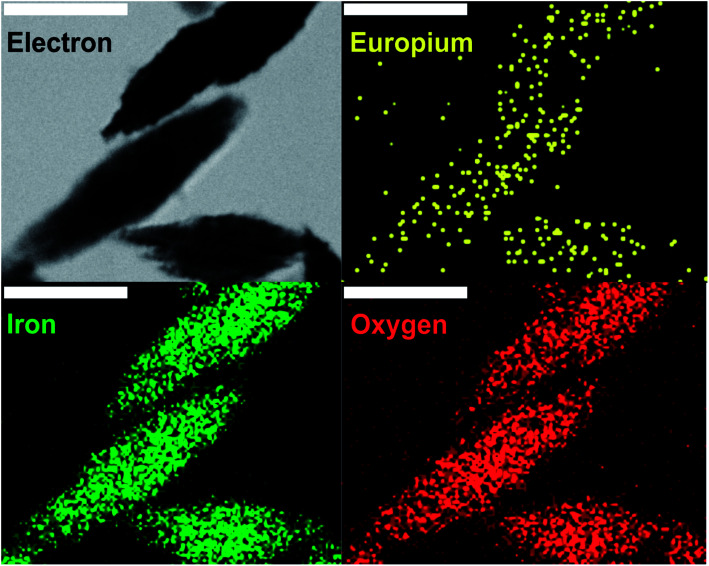
TEM-EDX mapping analysis of spindle-like europium-doped NPs. The data were collected for iron and oxygen (K series) and europium (L series). The scale bar is 100 nm.

The UV/Vis spectrum of NPs did not change significantly upon Eu doping (Fig. 1A), whereas the zeta potential of Eu-doped nanoparticles was negative (−11 ± 2 mV) in comparison to a positive value (15 ± 4 mV) for undoped iron oxide NPs. The change can be explained by a higher amount of nitrate anions coordinated to the surface of Eu-doped nanoparticles. That was also confirmed by spectroscopic Fourier Transform Infrared (FTIR) measurements (Fig. 1B) and in line with the more pronounced ability of europium to form anionic complexes with nitrate.^24^ As can be seen from the FTIR spectra of doped and undoped NPs, the amount of coordinated nitrate is higher in Eu-doped nanoparticles. That can be judged from the peaks at 1637–1300 cm^−1^,2854, and 2925 cm^−1^ (the similar peaks are also present in FTIR of pure europium nitrate [SDBS no. 40387]). Additionally, the spectra of both NPs samples contain two major peaks at 520, and 440 cm^−1^, which are characteristic for hematite [RRUFF ID: R040024.1], as well as three minor peaks at 3200, 900, and 805 cm^−1^ characteristic for hydrated iron oxides, *e.g.*, goethite [RRUFF ID: R050142.1]. The same results were obtained for the Raman spectra (Fig. 2C) of the NPs in comparison with the reported hematite spectrum,^25^ in which the peaks at 226, 292, 411, 498, and 612 cm^−1^ were similar to those at 222, 287, 402, 492, 604 cm^−1^ for undoped NPs and 222, 289, 402, 492, 608 cm^−1^ for Eu-doped NPs. Finally, the powder X-ray diffraction (XRD) studies (Fig. 2D) confirmed that the XRD patterns of Eu-doped NPs were similar to those of undoped NPs, which were in turn identical to the XRD pattern of hematite (JCPDS file no. 33-0664). The shift of the pattern to lower angles were observed for the XRD pattern of the doped NPs. Therefore, the cell parameters (Fig. 2E) for the europium-doped NPs slightly increased from *a* = 5.038(2) Å, *c* = 13.772(12) Å in hematite to *a* = 5.050(1) Å, *c* = 13.7869(3) Å. The change in cell parameters confirms the homogeneous europium doping of NPs with formation of only one phase slightly different from hematite. In addition, the doped NPs were studied using TEM-EDX mapping analysis; the analysis showed uniform europium distribution (Fig. 3).

**Fig. 4:**
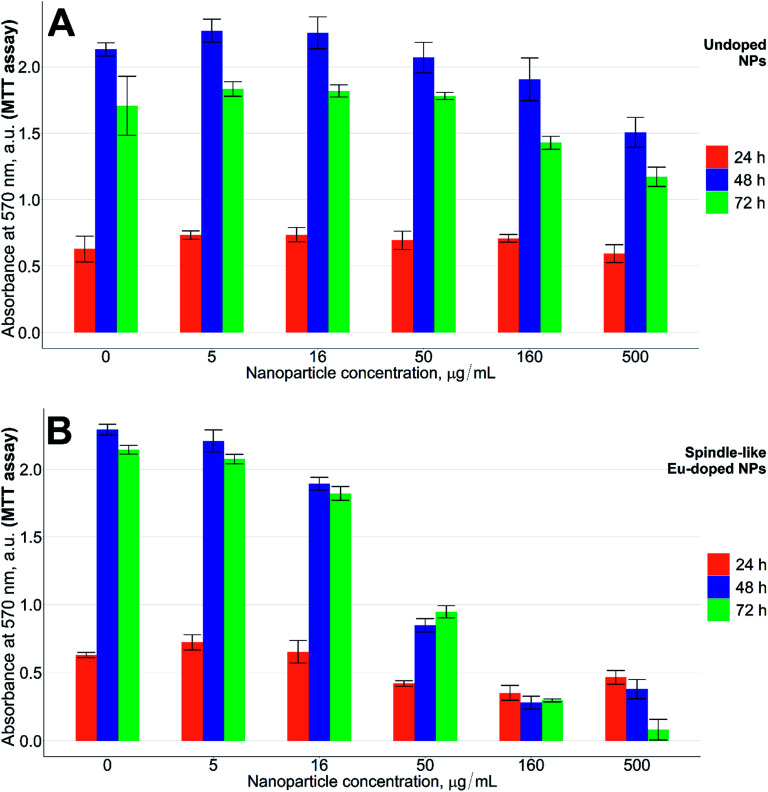
MTT test results. (A) Undoped NPs; (B) spindle-like Eu-doped NPs. The nanoparticles were incubated with CHO cells for 24 h (orange bars), 48 h (blue bars), and 72 h (green bars). The positive control of cells death was 1% Triton X-100 solution in medium. The data are presented as mean ± standard deviation (*n* = 6 per group).

Standard MTT assay was used to evaluate how the europium-doped iron oxide nanoparticles influenced cytotoxicity.^26,27,57^ As can be seen from Fig. 4, at concentrations above 50 μg mL^−1^ of the doped NPs, the CHO cells grow slower than the cells treated with undoped NPs at 24 h (*p* < 0.05). The difference is more profound at 48 h or 72 h (*p* < 0.001). Even at a concentration as high as 500 μg mL^−1^ of iron(iii) oxide NPs without europium, the cells grew at nearly the same rate as control cells.

The origin of toxicity of NPs could be heavy metal content, as well as a particular shape of the NPs. It is worth to mention that the nanoparticles elongated along one axis are reported to be more cytotoxic because of a larger contact area with a cell membrane^28–30^ and easier penetration of NPs through the cell membrane.^31^ A few reports on different europium-doped nanoparticles exist to date, however, most of them claim that the europium-doped nanoparticles show low to no toxicity and very good biocompatibility.^32–39^ A couple of publications report toxicity of the europium NPs at high concentrations and high content of europium.^40–42^ Most studies describe that cell death from the Eu-contained NPs could be caused by accumulation of NPs in lysosomes followed by cell rupture and necrosis,^43^ by wrinkling of cells shape and shrinking the nuclei,^44^ by stopping cell proliferation through the down-regulation of the Ca^2+^ relying proteins, and by an increase in reactive oxygen species (ROS) concentration and mitochondrial damage.^45^

**Fig. 5:**
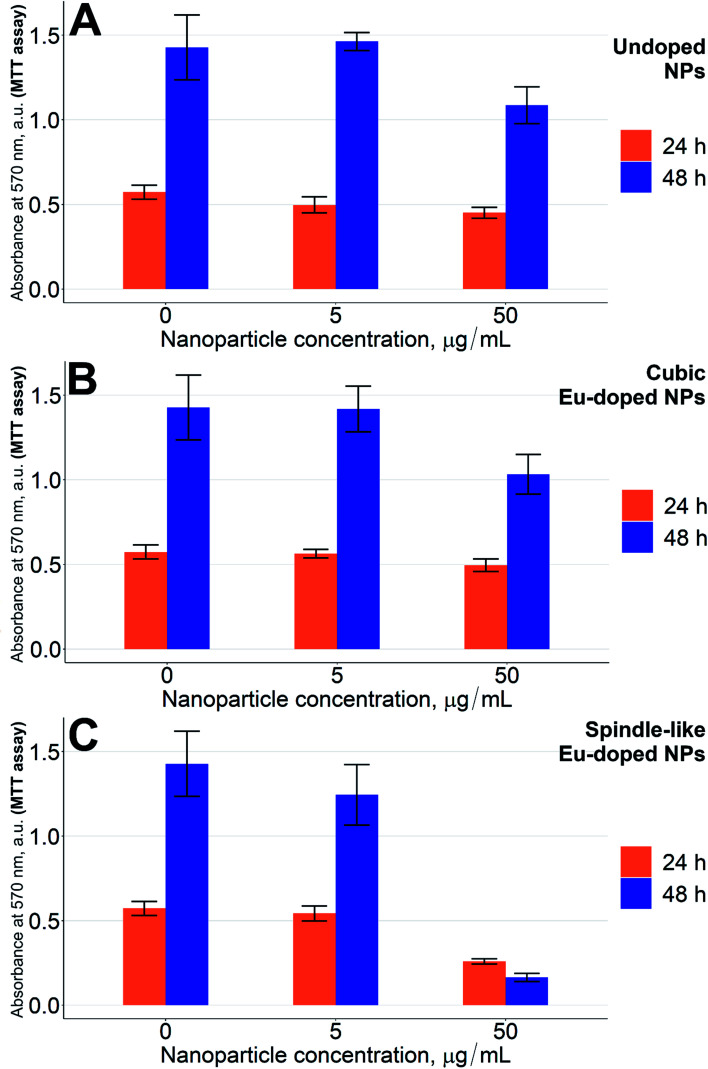
MTT test results. (A) Undoped NPs; (B) cubic Eu-doped NPs; (C) spindle-like Eu-doped NPs. The nanoparticles were incubated with CHO cells for 24 h (orange bars), 48 h (blue bars). The positive control of cells death was 1% Triton X-100 solution in medium. The data are presented as mean ± standard deviation (*n* = 6 per group).

Since the low concentration of europium (2.4%) could not alone induce severe cytotoxicity, the toxic effects of our NPs should be due to a combination of both factors, where the shape increases the toxicity of low levels of europium in NPs. To supplement the hypothesis on the shape-induced toxicity of the NPs, another MTT test was carried out using the lowest active concentrations of the spindle-like NPs (50 μg mL^−1^) together with the cubic europium-doped NPs of the same europium content (that we show in Fig. 1B). Indeed, as can be seen from Fig. 5, the growth of cells in the presence of cubic europium-doped is similar to that of the cells treated with undoped iron oxide NPs (Fig. 5B and A, respectively), whereas the growth of cells treated with spindle-like europium-doped NPs is slower (*p* < 0.01) (Fig. 5C).

**Fig. 6:**
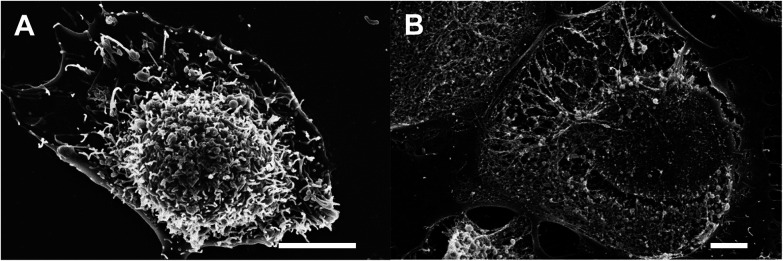
SEM images of CHO cells. (A) An intact cell; (B) cell after treatment with spindle-like Eu-doped NPs with the destructed membrane. The scale bar is 2 μm.

**Fig. 7:**
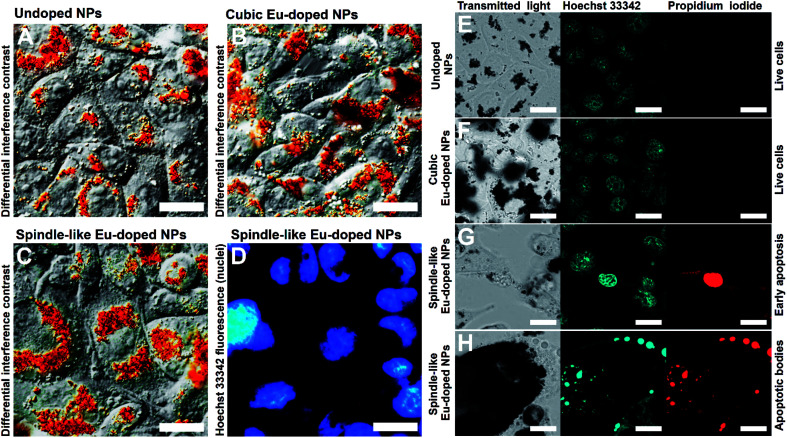
Optical images of incubated with NPs CHO cells. (A–C) Collected using DIC microscopy images; (D) acquired *via* fluorescence microscopy image; (E–H) acquired *via* laser scanning fluorescence microscopy images. In all experiments cells were incubated with nanoparticles for 48 h. In images A, B, and C cells were incubated with undoped NPs, Eu-doped cubic NPs, and Eu-doped spindle-like NPs respectively. Image D shows fluorescence of Hoechst-stained cells from image C. Images (E–H) demonstrate cells incubated with undoped NPs (E), Eu-doped cubic NPs (F), and Eu-doped spindle-like NPs (G and H), stained with Hoechst and PI dyes. The scale bar is 20 μm.

Additional studies of the effect of the spindle-like NPs on CHO cells were carried out using microscopic techniques such as SEM and optical microscopy. SEM study showed that the CHO cells after treatment with the spindle-like NPs were damaged (Fig. 6B and S1†). The destruction of the cell membrane was shown, and the fibrillar components of the cytoskeleton, and membrane organelles were visible. To observe interactions between the NPs and living cells, the differential interference contrast (Fig. 7A–C) and fluorescent microscopy (Fig. 7D) were used. Fig. 7C and D clearly show that the nanoparticles did not penetrate into the cell nuclei. Additionally, the cells treated with NPs for 48 h were stained with Hoechst and PI (cell-impermeable DNA dye, stains DNA if cell membrane is permeabilized) to visualize live, necrotic and apoptotic cells. Laser scanning confocal microscopy (Fig. 7E–H) showed that treatment with undoped iron oxide NPs and cubic europium-doped NPs did not cause any observable signs of apoptosis and necrosis (Fig. 7E and F). The Hoechst staining showed that cells had round-shaped and evenly-stained nuclei. Moreover, the PI-stained cells were not found; this observation is in an agreement with the fact that live cells have a non-permeabilized membrane. On the other hand, the fragmented and PI-stained nuclei (Fig. 7G) and apoptotic bodies (Fig. 7H) were observed in the cells treated with spindle-like europium-doped NPs.^46^

**Fig. 8:**
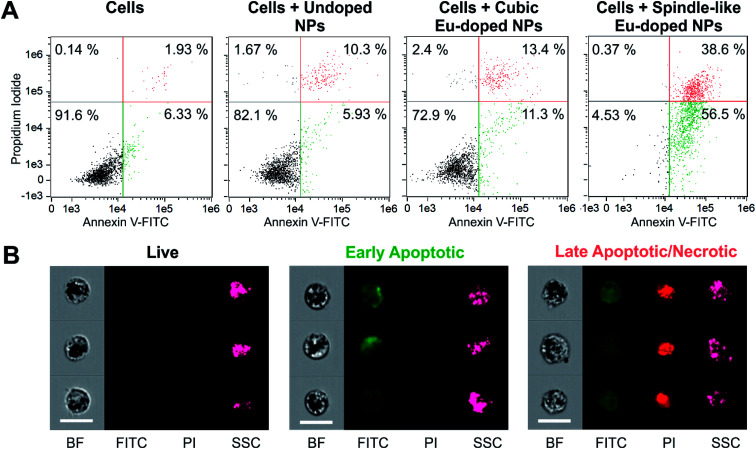
Imaging flow cytometry analysis of seeded for 48 h CHO cells with different NPs. The cells were stained with PI and FITC-labeled annexin V (Annexin V-FITC). (A) Compensated dot plots of PI/Annexin V-FITC staining of the cells after the incubation with NPs. Lower left, lower right, and higher right areas include viable, early apoptotic, and late apoptotic/necrotic subpopulations, respectively. The numbers in areas demonstrate the share of the subpopulation in all collected cells. (B) Representative cell images from all subpopulations. The images are acquired in bright field (BF), FITC-sensitive, PI-sensitive, and side scatter (SSC) channels. The scale bar is 20 μm.

To investigate this behavior in a quantitative manner, we examined the NPs-associated effects on cells with imaging flow cytometry analysis.^58^ FITC-labeled Annexin V and PI were used to identify live, apoptotic and necrotic cells. Annexin V binds to phosphatidylserine that translocates to the external leaflet of the plasma membrane during apoptosis. PI is a membrane-impermeable nucleic acid intercalator: early apoptotic cells exclude PI, while late apoptotic, and necrotic cells stain positively. The dot plot for cells after 48 h incubation with the spindle-like europium-doped NPs indicated that less than 5% of the cells were alive (Fig. 8), while in other samples the populations of living cells were approximately between 70% and 90%. Thus, the experiments have confirmed that the spindle-like NPs doped with europium have a pronounced cytotoxic effect and cause early apoptosis (*ca.* 57%) and late apoptosis/necrosis (*ca.* 39%) of cells.

**Fig. 9:**
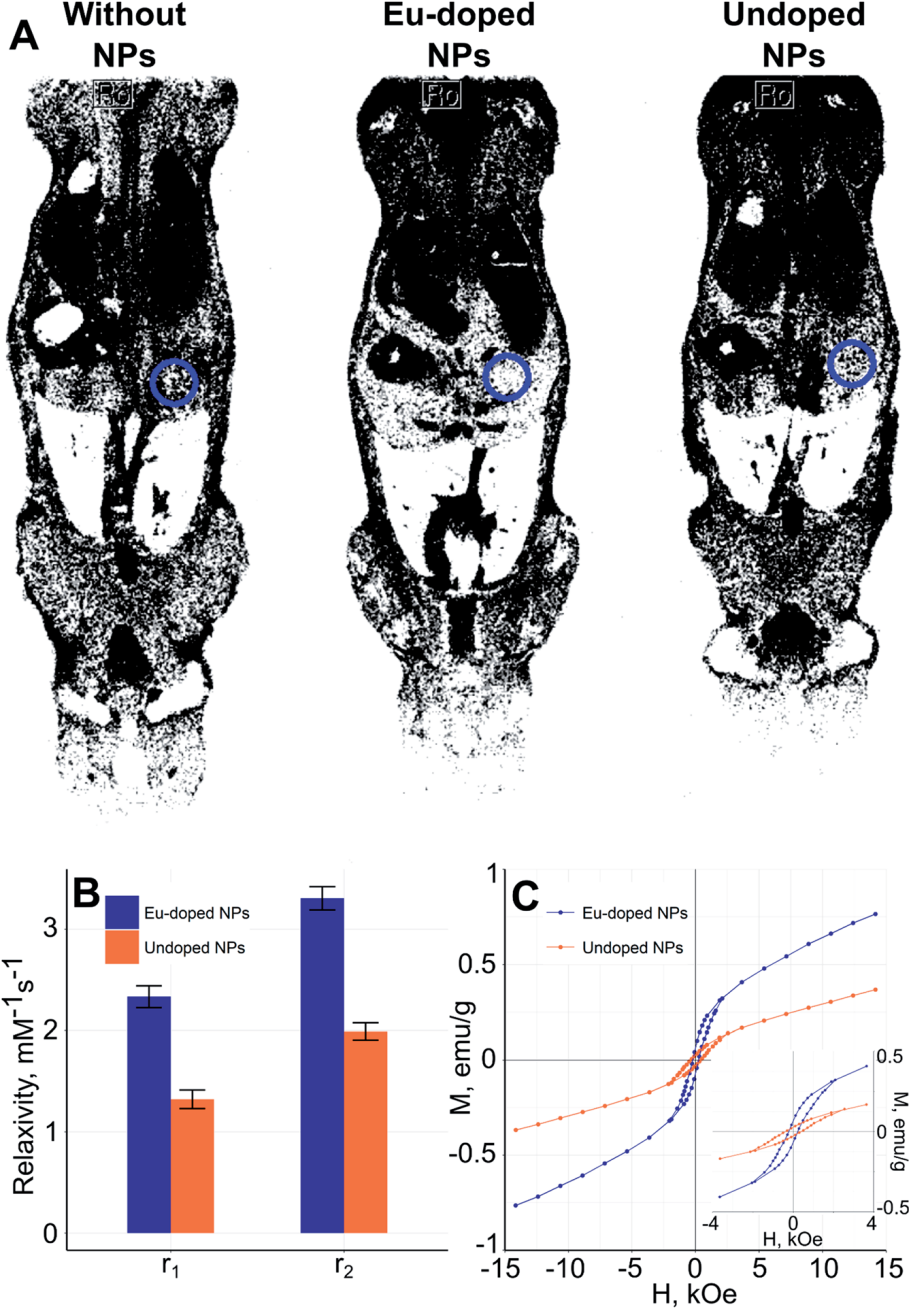
Inverted MRI images of mice and magnetic properties of the NPs. (A) Images of injected and noninjected mice (RARE sequence), repetition time = 300 ms, echo time = 10 ms, circles indicate contrasted liver regions; (B) relaxivities of the NPs at RT (measured at external field of 0.23 T), (C) magnetization curves of the NPs at RT.

The paramagnetic nature of the obtained particles was verified by the magnetic particle quantification (MPQ) technique,^47^ which showed no presence of ferro- and superparamagnetic fractions at the level of 0.1 ng mL^−1^. *In vivo* evaluation of the MRI-contrasting properties of the NPs was performed in an animal model using the tomography scanner. The NPs were coated with CMD to ensure colloidal stability of the NPs. Each animal was scanned in various modes with the different TR (repetition time – duration between the corresponding consecutive points on a repeating series of pulses and echoes) and TE (echo time – period between the middle of the first pulse to the middle of echo) parameters. The measurements were carried out using the FLASH (Fast Low Angle Shot) and the RARE (Rapid Acquisition with Refocused Echoes) sequences. For estimation of the contrasting properties of the NPs, we used intensities of the MRI signal in the liver area of the MRI images of test and control mice. To remove the error associated with the individual imaging parameters and peculiarities of the animal under test, all the measured MRI signal intensities were compared to the averaged intensity of the muscle tissue signal, which was almost independent of the NPs presence. The contrast factor was calculated as *K* = *I*_muscle_/*I*_liver_, where *I*_muscle_ and *I*_liver_ were intensities of the MRI signals from muscle and liver areas in the mouse, respectively. Because of the bigger *I* the brighter the chosen area, the bigger *K* (Table S1†) indicate the more pronounced negative contrast of NPs. Fig. 9A and Table S1† illustrate that the NPs doped with europium and undoped iron oxide NPs offer MRI contrasting properties in FLASH and RARE (Fig. S2†) modes of scanning. Negative contrasting properties of europium-doped NPs were also supported by higher values of both *r*_1_ and *r*_2_ relaxivities (Fig. 9B). Also, the europium-doped NPs are more susceptible to the field (Fig. 9C); the magnetization curves are typical for nano- and microsized hematite.^48,49^ The changes of the magnetic properties caused by lanthanide doping is often observed for the iron oxide NPs of different composition and size. The primary use of such phenomenon is enhancement of the contrasting properties of NPs.^50–53^

**Fig. 10:**
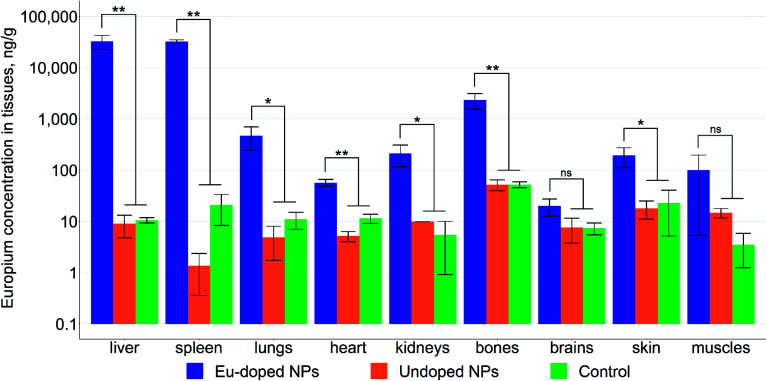
Concentration of europium in mice organs according to ICP-MS. Statistical significance was determined using ANOVA with Tukey's post-hoc test. The data are presented as mean ± standard deviation (*n* = 3 per group).

As can be seen from Fig. 9A the nanoparticles are negative MRI contrast agents, concentrating in the liver and spleen. The ICP-MS analysis (Fig. 10) of acid-digested mice organs has shown that NPs concentration in liver and spleen exceeds by at least an order of magnitude that in the rest tissues. In addition, the leaky endothelial wall in liver, spleen, and bone marrow leads to the maximization of NPs concentration in the corresponding organs; this fact allows us to conclude that the NPs could accumulate in a solid tumor due to the “enhanced permeability and retention” (EPR) effect.^54^ Additionally, the analysis revealed that more than 98% of the NPs were indeed accumulated in liver (78.4%) and spleen (20.4%). This finding is in accordance with the fact that the iron oxide nanoparticles of size between 10 nm and 100 nm are cleared from the bloodstream by the mononuclear phagocyte system (MPS) mostly located in liver and spleen.^55^

For additional evaluation of the cytotoxic properties of NPs, we have studied the biochemical parameters in serum 2 h after injection of 1 mg of the NPs (Table S2†). The liver damage was evaluated by the levels of alanine aminotransferase (ALT), aspartate aminotransferase (AST) and bilirubin (BIL). The nephrotoxicity was determined by creatinine (CT), and the heart damage – by lactate dehydrogenase (LDH) enzyme. We observed a decrease in the level of AST and BIL in the case of europium-doped nanoparticles, and a growth in the level of ALT in the group injected with the undoped iron oxide NPs. At the same time, kidneys and heart were not significantly affected. Although all enzymes were in the normal clinical range, this study showed that nanoparticles make a greater impact on the liver than the other organs.

## Conclusions

We have demonstrated a novel facile method for preparation of the europium-doped iron oxide nanoparticles. According to the MTT test, imaging flow cytometry, optical and electron microscopy, the NPs possess high cytotoxicity, which, in spite of low europium content, is due not only to the influence of heavy metal but also to the shape of the NPs. Therefore, this is an example of cytotoxic NPs with low heavy metal content. Various iron oxide particles are already approved in the clinic for MRI-contrasting and iron deficiency anemia treatment. The development of our Eu-doped NPs is highly beneficial because they have both MRI-imaging and cytotoxic modalities. This allows early efficient evaluation of NPs-based therapy by facile noninvasive measurement of NPs delivery to tumors. Therefore, the NPs are promising as cytotoxic and MRI-contrasting components for development of novel nanoagents for theranostic applications.

## Conflicts of interest

There are no conflicts to declare.

## Supplementary Material

RA-010-C9RA10683A-s001
