# Supplementary material for: Spindle-like MRI-active europium-doped iron oxide nanoparticles with shape-induced cytotoxicity from simple and facile ferrihydrite crystallization procedure
Source: RSC Adv. 2020 Feb 18;10(12):7301–12. doi: 10.1039/c9ra10683a (PMC9049874; doi:10.1039/c9ra10683a)
Supplement: RA-010-C9RA10683A-s001 [file RA-010-C9RA10683A-s001.pdf]

Electronic Supplementary Material (ESI) for RSC Advances.
This journal is © The Royal Society of Chemistry 2020

Electronic supplementary information (ESI) for

Spindle-like MRI-active europium-doped iron oxide nanoparticles with shape-induced cytotoxicity from simple and facile ferrihydrite crystallization procedure

Afanasy V. Lunin,[a] Ilya L. Sokolov,[a] Ivan V. Zelepukin,[a,b] Ilya V. Zubarev,[a] Maria N. Yakovtseva,[a] Elizaveta N. Mochalova,[a,c] Julian M. Rozenberg[a], Maxim P. Nikitin,[a,b] and Eugene L. Kolychev[a,c,]*

[a.] Moscow Institute of Physics and Technology (National Research University), 9 Institutskiy per., Dolgoprudny, Moscow Region, 141700, Russia.
[b.] Shemyakin–Ovchinnikov Institute of Bioorganic Chemistry, Russian Academy of Sciences, Ulitsa Miklukho-Maklaya, 16/10, Moscow, 117997, Russia.
[c.] Prokhorov General Physics Institute of the Russian Academy of Sciences, 38 Vavilov St., Moscow 119991, Russia.

E-mail: eugene.kolychev@gmail.com

$$Percent\ \ of\ \ injected\ \ dose\ \ per\,organ\ [\%] = \frac{Europium\ \ organ\ \ concentration\ [\mu g/g] \times Organ\ \ weight\ \ [g]}{Injected\ \ europium\ \ mass\ [\mu g]} \times 100\ \%$$

Formula S1. Calculation of NPs retained by organs. Europium organ concentration and Injected europium mass were measured using ICP-MS. Organ weight was measured using precision balances.

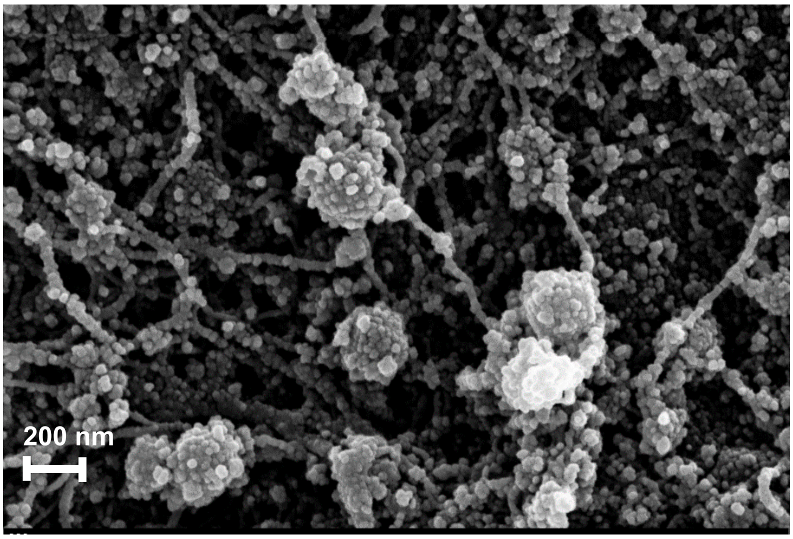

Figure S1. SEM of part of the cytoplasm of a CHO cell after treatment with the NPs. The cytoskeleton network and vesicles associated with microtubules are visible. The plasma membrane is destroyed.

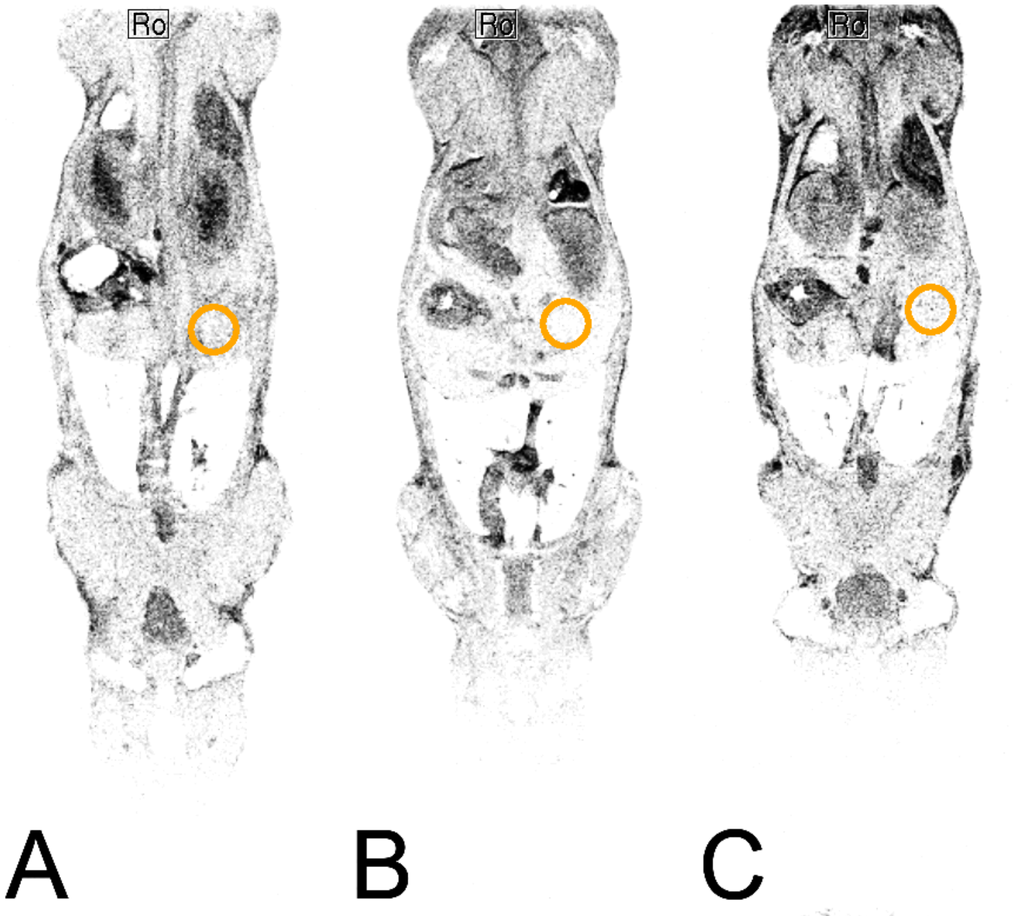

Figure S2. Inverted MRI Images (RARE)
A –control mice, B- doped with Eu NPs, C- pure iron oxide NPs

Table S1. Results of contrasting properties estimate.

	Europium NPs	Pure Iron oxide NPs	control
K (FLASH, TR 300ms, TE10ms)	4.07	3.49	1.04
K (RARE, 3000ms, 40ms)	1.20	1.10	0.99

Table S2. Biochemical assay in mice serum. ALT, alanine aminotransferase; AST, aspartate aminotransferase; BIL, bilirubin; CT creatinine; LDH, lactate dehydrogenase.

Group	ALT (mmol/(h*L))	AST (mmol/(h*L))	BIL (mmol/L)	CT (umol/L)	LDH (U/L)
Eu	0.95±0.19	0.43±0.04	0.25	141.1	276±89
NEu	1.31±0.07	0.62±0.02	3.14	160.5	276±121
Control	0.73±0.31	0.6±0.01	2.71	135.6	196±67
